# Barriers and facilitators to the integration of mental health services into primary health care: a systematic review

**DOI:** 10.1186/s13643-018-0882-7

**Published:** 2018-11-28

**Authors:** Edith K. Wakida, Zohray M. Talib, Dickens Akena, Elialilia S. Okello, Alison Kinengyere, Arnold Mindra, Celestino Obua

**Affiliations:** 1 Department of Psychiatry, Mbarara University of Science and Technology, Mbarara, Uganda; 2 Department of Medical Education, California University of Science and Medicine, California, USA; 3 Africa Center for Systematic Reviews and Knowledge Translation, College of Health Sciences Makerere University, Kampala, Uganda; 4 Department of Psychiatry, Makerere University, Kampala, Uganda; 5 Library, Africa Center for Systematic Reviews and Knowledge Translation, College of Health Sciences, Makerere University, Kampala, Uganda; 6 Office of Research Administration, Mbarara University of Science and Technology, Mbarara, Uganda; 7 Department of Pharmacology and Therapeutics, Mbarara University of Science and Technology, Mbarara, Uganda; 8 Mbarara University of Science and Technology, Mbarara, Uganda

**Keywords:** Integration, Mental health services, Primary health care, Barriers and facilitators, Systematic review

## Abstract

**Background:**

The objective of the review was to synthesize evidence of barriers and facilitators to the integration of mental health services into PHC from existing literature. The structure of the review was guided by the SPIDER framework which involves the following: *Sample* or population of interest—primary care providers (PCPs); *Phenomenon of Interest*—integration of mental health services into primary health care (PHC); *Design*—influenced robustness and analysis of the study; *Evaluation*—outcomes included subjective outcomes (views and attitudes); and *Research type*—qualitative, quantitative, and mixed methods studies.

**Methods:**

Studies that described mental health integration in PHC settings, involved primary care providers, and presented barriers/facilitators of mental health integration into PHC were included in the review. The sources of information included PubMed, PsycINFO, Cochrane Central Register of Controlled trials, the WHO website, and OpenGrey. Assessment of bias and quality was done using two separate tools: the Critical Appraisal Skills Program (CASP) qualitative checklist and the Effective Public Health Practice Project Quality Assessment Tool for Quantitative Studies.

**Results:**

Twenty studies met the inclusion criteria out of the 3353 search results. The most frequently reported barriers to integration of mental health services into PHC were (i) attitudes regarding program acceptability, appropriateness, and credibility; (ii) knowledge and skills; (iii) motivation to change; (iv) management and/or leadership; and (v) financial resources. In order to come up with an actionable approach to addressing the barriers, these factors were further analyzed along a behavior change theory.

**Discussion:**

We have shown that the integration of mental health services into PHC has been carried out by various countries. The analysis from this review provides evidence to inform policy on the existing barriers and facilitators to the implementation of the mental health integration policy option. Not all databases may have been exhausted.

**Systematic review registration:**

PROSPERO 2016 (Registration Number: CRD42016052000) and published in BMC Systematic Reviews August 2017.

**Electronic supplementary material:**

The online version of this article (10.1186/s13643-018-0882-7) contains supplementary material, which is available to authorized users.

## Introduction

### Rationale

Mental health is a state of well-being in which every individual realizes their own potential, copes with the normal stresses of life, works productively and fruitfully, and is able to make a contribution to their community [1]. When one is unable to function to their full life in society, because of conditions that affect cognition, emotion, and behavior, they are said to have mental illness [2]. Mental health is an integral part of health; however, health systems have not been able to adequately respond to the burden of mental health. Up to 85% of people with severe mental illness in low- and middle-income countries (LMIC) receive no treatment for their disorder [3, 4]. Mental and behavioral disorders are estimated to account for 14% of the global burden of disease with sub-Saharan Africa (SSA) accounting for 19% of the burden [5]. If untreated, mental and behavioral disorders are likely to cause severe disability and heavy socio-economic burden on families and communities [6–10]. Integrating mental health services into primary health care (PHC) is among the most viable means of closing the treatment gap and ensuring that people get the mental health care they need [8, 10].

The PHC setting is the first point of contact an individual has with the health system and is essential to making health care universally accessible to individuals and families in the community in an acceptable and affordable way, with their full participation [11, 12]. The concept of PHC was formally adopted by the World Health Organization (WHO) through the Alma-Ata declaration as the preferred method for providing a comprehensive, universal, equitable, and affordable healthcare service [11], and it had the ability to reduce stigma, improve access to care, reduce chronicity of mental illness, and improve social integration [5, 12, 13]. The Alma-Ata model of mental health integration recommends that countries build or transform their mental health services to (i) promote self-care, (ii) build informal community care services, (iii) build community mental health services, (iv) develop mental health services in general hospitals, and (v) limit reliance on psychiatric hospitals [14].

Furthermore, evidence shows that mental health care can be delivered effectively in PHC settings and that once identified, most mental illnesses can be treated using cost-effective means [10, 15, 16]. Treatment of common mental disorders at PHC can be improved through collaborative care interventions that yield better access to care, physical as well as mental health outcomes, and improved overall cost-effectiveness [17, 18].

The past decades have seen enormous investment by the WHO in ensuring that mental health services are integrated into PHC. The WHO issued key recommendations [5] to guide the process include the following: (i) conducting a preliminary situational analysis of the best options for the treatment and care of mental disorders at the different levels of care; (ii) building on existing networks/structures and human resources to provide mental health services; (iii) re-distributing funding from tertiary to secondary and primary levels of care, making new funds available; (iv) delineation of mental disorders to be treated at the primary care level; (v) training of primary care staff in identification and treatment of mental disorders; (vi) recruitment and/or education of new primary care providers (PCPs); (vii) availing basic psychotropic medicines at primary and secondary care levels; and (viii) adequate supervision and support of PCPs by mental health specialists for a successful integration.

Integration of mental health into PHC has been carried out in various countries and in different forms [19–23]. However, evidence shows inadequate or lack of integration of mental health services into PHC due to a number of factors [24–26], which have not been summarized and availed to relevant stakeholders (PCPs, policy makers, and the WHO) for re-evaluation. Using the SURE (Supporting the Use of Research Evidence) framework [27] as the candidate framework to categorize the barriers and facilitators, the SPIDER (Sample, Phenomenon of Interest, Design, Evaluation, Research type) framework [28] to structure the review, and the “best fit” approach [29] in the synthesis of the data, the objective of this was to synthesize evidence of barriers and facilitators to the integration of mental health services into PHC from existing literature.

## Methods

The protocol for this review was registered with PROSPERO 2016 under Registration Number: CRD42016052000 and subsequently published in BMC Systematic Reviews journal in 2017 [30].

The review was conducted according to the Preferred Reporting Items for Systematic Reviews and Meta-Analyses (PRISMA) checklist recommended for systematic reviews [31], as outlined in Additional file 1, and the results were analyzed following the “best fit” framework synthesis. The “best fit” framework involves the following steps: (a) the systematic identification of relevant primary research studies using the SPIDER approach, (b) identification of relevant publications using a search strategy (Additional file 2), (c) extraction of data on the characteristics of included studies and their appraisal for quality, (d) coding of evidence from included studies against an a priori framework (SURE framework), (e) creating new themes by performing thematic analysis on any evidence that cannot be coded against the a priori framework, (f) producing a new framework composed of a priori and new themes supported by evidence, and (g) revisiting evidence to explore relationships between themes. We did not create new themes as anticipated in our protocol [30], as all the identified factors fitted within the a priori framework during the coding process. We created a model combining the SURE domains, identified barriers/facilitators, and the capability opportunity and motivation framework (COM-B) [32] in order to understand the PCP behavior towards implementation of the integration policy option.

The SURE framework, developed for implementing health changes within Africa, has 28 domains (Additional file 3) that we found applicable in this review. These domains are categorized at five levels: (a) recipients of care, (b) providers of care, (c) other stakeholders, (d) health systems constraints, and (e) social and political constraints. Under providers of care, we were interested in awareness of guidelines on integration of mental health services into PHC, knowledge about mental health disorders, and training in implementation of the guidelines. We further looked for perceptions about integration of mental health services into PHC and factors that motivate PCPs to take on new tasks. When considering health systems constraints, we were interested in identifying how the domains affect the PCP’s ability to integrate mental health services into PHC. Subsequently, we look at how social and political constraints affect integration of mental health services into PHC.

The COM-B theory postulates that in order to change behavior of PCPs to implement mental health services in PHC, one needs to change one or more of “capability” to perform the behavior and/or “opportunity” and “motivation” to carry out the behavior. *Capability* is the individual’s psychological and physical capacity to engage in the activity concerned. *Opportunity* is all the factors that lie outside the individual that make the behavior possible or prompt it. *Motivation* is all those brain processes that energize and direct behavior, not just goals and conscious decision-making; it includes habitual processes, emotional responding, and analytical decision-making [33].

The “best fit” framework synthesis is commonly used for qualitative and mixed methods studies; however, in this review, we also included articles that used only quantitative methods in order to accommodate studies that quantified barriers and facilitators to integration of mental health service.

### Search strategy

A systematic search of literature was performed in February 2017, and updated in June 2017, using a search strategy that was developed by AK, a qualified librarian on the review team, and peer-reviewed by DK, a mental health specialist. We searched three databases including PubMed, PsycINFO, and Cochrane Central Register of Controlled trials for eligible studies. In the protocol, we indicated that EMBASE would be included among the databases to be searched; however, due to accessibility limitations, it was not searched hence potentially limiting our findings. We also searched for gray literature from the WHO website and OpenGrey. The search terms were kept broad, and no date restriction was placed in order to capture potentially eligible studies. Medical Subject Headings (MeSH) terms, keywords, and their synonyms were used to develop the search strategy for PubMed and adapted for the other databases (Additional file 2). Following the literature search, the references were exported to an EndNote database (EndNote version X7.7.1 Thomson Reuters) and the duplicates removed.

### Inclusion and exclusion criteria

Selection of articles was based on the SPIDER (Sample, Phenomenon of Interest, Design, Evaluation, Research type) framework, chosen because of its suitable application to qualitative and mixed methods research. We included articles whose *Sample* or population of interest were PCPs, CHWs, healthcare managers, and policy makers who had been involved in the integration of mental health into PHC in general health care, collaborative care, and/or specialized health care in any country. The *Phenomenon of Interest* was integration of mental health services into general health care, delivered at primary or community healthcare settings, and collaborative in nature (the PCPs, CHWs, and healthcare managers, working together). The study *Design* influenced the robustness and analysis of the study. For *Evaluation*, outcomes included subjective outcomes such as views and attitudes [28]. For *Research type*, the review covered three research types: (i) qualitative studies that used cappropriate methods of data collection and analysis (such as ethnography, grounded theory, phenomenology, case studies) [34–36], (ii) quantitative studies, and (iii) mixed methods—studies combining qualitative and quantitative methods of data collection and analysis which included cross sectional studies, case-control studies, cohort studies, quasi-experimental studies, and randomized control trials. Articles that were not specifically in a PHC or community setting were excluded.

### Data extraction and management

The full text of all the papers that were identified as potentially relevant were retrieved by AK and DA and double-screened by EW and AM to ensure that they were eligible for inclusion before data extraction. All disagreements were resolved by consensus and/or discussion with the senior reviewer CO. Results of the full text were shared with the remaining review authors to validate their eligibility, and there was no dispute. Articles which met the inclusion criteria following a full-text review by EW and AM were selected for data extraction and synthesis. The characteristics of studies included the authors and year of publication, the study title and type, the country of study, the study setting and facility type, the study population, and barriers/challenges and/or facilitators/enablers to the integration of mental health services into PHC (Additional file 4).

### Risk of bias and quality assessment

Assessment of bias and quality was done by the primary author (EW) and AM in consultation with EO, ZT, and CO using two separate tools: the Critical Appraisal Skills Program (CASP) qualitative checklist [37] and the Effective Public Health Practice Project (EPHPP) quality assessment tool for quantitative studies [38]. The CASP checklist consisted of 10 questions; the first two questions were screening questions, and if the answers to both were “yes,” it was worth proceeding with the remaining questions. The EPHPP tool consisted of eight component ratings, which were classified using the parameters “yes,” “no,” “cannot tell,” or “not applicable.” “Yes” corresponded to strong, “no” moderate, and “cannot tell” weak. These checklists were used because they have comprehensive instructions which enabled the authors to assess the relevance and rigor of all included studies [39] (Additional file 5). In order to limit publication bias, articles from both published and gray literature were included.

### Data synthesis

Data synthesis was synergistically done combining qualitative, quantitative, and mixed methods studies. The following key variables were extracted from the articles by EW and AM to ensure inter-rater reliability: (i) lead author, (ii) year of publication, (iii) country and setting of the study, (iv) study aim, (v) study design, (vi) facility type, (vii) participants/sample size, (viii) data collection method, (ix) mental health type, and (x) barriers and or enablers (Additional file 4). The identified barriers and facilitators were then coded using an a priori framework and compared to determine the most frequently reported barriers and facilitators to the integration of mental health services into PHC.

## Results

### Study selection

The electronic search yielded a total of 3144 studies from PubMed (*n* = 2435), PsycINFO (*n* = 291), and Cochrane Central Register of Controlled trials (*n* = 418) and an additional 209 studies from the WHO website (*n* = 192) and OpenGrey (*n* = 17), giving an overall total of 3353 articles. After removing 98 duplicates, 3255 studies remained for screening. The screening based on title and abstract resulted in the exclusion of 3229 articles with the main reasons for elimination being that the studies were either not conducted in a PHC setting or they had nothing to do with integration of mental health services and/or the intervention was not related to service provision. Of the 28 potentially eligible studies, full-text screening led to a further exclusion of eight studies, which were deemed not relevant to the study aim. The excluded studies were not conducted in the general population, but rather, they were conducted among specific populations such as veterans only or in mental healthcare settings and did not consider PCPs’ barriers and/or facilitators to integration of mental health services into PHC.

### Study characteristics

**Fig. 1:**
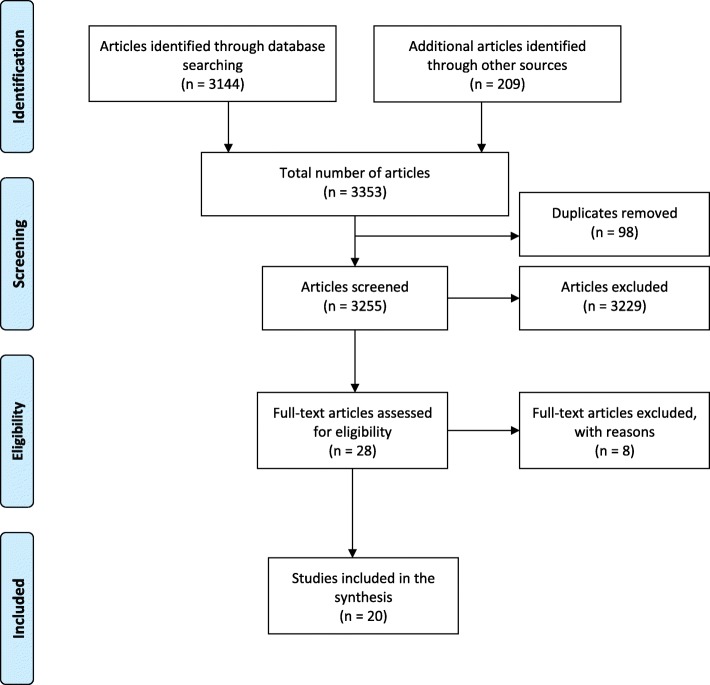
PRISMA flow diagram of research studies search

Finally, 20 studies were included in the synthesis (Fig. 1), composed of 12 qualitative, 4 quantitative, and 4 mixed methods studies (Table 1). Analysis of the quantitative and mixed methods studies was descriptive, while that of qualitative studies was thematic [39]. Six studies were conducted in the USA [40–45], two in Australia [19, 46], and one in each of the following countries: India [47], Israel [26], Ethiopia [24], Saint Vincent and the Grenadines (SVG) [48], Zambia [49], Nigeria [50], Mexico [51], Brazil [52], Zimbabwe [53], Kenya [54], Uganda [55], and UK [56]. Of the listed countries, ten were developing countries [24, 47–55] with the majority [24, 49, 50, 53–55] being in Sub-Saharan Africa, and ten were developed countries [19, 26, 40–46, 56] according to the United Nations country classification [57] (Table 1). Fourteen articles reviewed described both barriers and facilitators to the integration of mental health services into PHC [19, 24, 40, 42, 44, 45, 47–50, 52, 53, 55, 56], while six described only barriers [26, 41, 43, 46, 51, 54]. Most articles were relevant to nurses [19, 24, 40, 42, 44–46, 48–51, 53–56], while three were exclusively to doctors (general practitioners and specialties) [26, 41, 47] and another three were relevant to CHWs [40, 46, 50]. The articles reviewed were published between 2007 and 2017.

**Table 1:** Studies included in the synthesis

References	First author	Study type	Location	Setting
[19]	Barraclough F	Qualitative	Australia	Developed
[26]	Ayalon L	Qualitative	Israel	Developed
[54]	Jenkins R	Qualitative	Kenya	Developing
[56]	Knowles SE	Qualitative	UK	Developed
[40]	Mesidor M	Qualitative	USA	Developed
[42]	Fickle JJ	Qualitative	USA	Developed
[46]	Henderson J	Qualitative	Australia	Developed
[43]	Henke RM	Qualitative	USA	Developed
[44]	Hill SK	Qualitative	USA	Developed
[55]	Kigozi FN	Qualitative	Uganda	Developing
[51]	Martinez W	Qualitative	Mexico	Developing
[45]	Zubkoff L	Qualitative	USA	Developed
[47]	Cowan J	Quantitative	India	Developing
[50]	Mosaku KS	Quantitative	Nigeria	Developing
[41]	Davis DW	Quantitative	USA	Developed
[49]	Kapungwe A	Quantitative	Zambia	Developing
[24]	Abera M	Mixed methods	Ethiopia	Developing
[52]	Athie K	Mixed methods	Brazil	Developing
[53]	Duffy M	Mixed methods	Zimbabwe	Developing
[48]	Winer RA	Mixed methods	Saint Vincent and the Grenadines (SVG)	Developing

### Quality of articles included

All the qualitative studies had a clear statement of the aims, research designs, and recruitment strategies appropriate to address the purpose of the research. The studies showed sufficient rigor in data analysis with a clear statement of findings and value of the research, save for one study [24], which did not show substantive rigor in data analysis when compared to the other studies. The quantitative studies on the other hand had varying methodological differences [39] according to the Effective Public Health Practice Project Quality Assessment Tool. Although the included studies had representative target populations selected to participate, only three studies [24, 47, 50] showed the percentage of selected individuals who agreed to participate in the studies. All the studies indicated the study design—three of which [40, 54, 56] had qualitative studies conducted within randomized trials. No study reported on confounders, withdrawals or dropouts, and the intervention integrity; however, two studies indicated blinding in the trials [40, 54]. Additionally, all the included studies reported the data collection methods although the unit of allocation and unit of analysis were either not reported or not applicable.

### Barriers and facilitators to the integration

We have summarized the barriers and facilitators to integration of mental health services into PHC from the chosen articles in Table 2. The barriers and facilitators were categorized along the SURE framework and divided according to domains related to *providers of care* and *health system constraints* to highlight the different levels at which the factors (barriers and facilitators) may occur. We report on only the most frequently cited factors.

**Table 2:** Levels, domains, barriers, and facilitators to integration

Level	SURE framework concepts	Barriers	Facilitators
Providers of care	Knowledge and skills	Inability to diagnose and treat mental illnesses	• Perceived competence in mental health care • Knowledge of mental disorder symptoms • Prior training in mental health
		Inability to identify either an antipsychotic or antidepressant medication	
		Lack of knowledge regarding psychosocial interventions	
		Inadequate training in the use of mental health screening tools	
		Inadequate training in current evidence-based treatment	
		Limited mental health awareness in the community	
		Lack of knowledge about health system structures	
		Lack of knowledge about processes for management of mental health	
	Attitudes regarding program acceptability, appropriateness, and credibility	Beliefs that mental illness is a strange behavior	• Agreement that mental health problems are common and need to be attended to • Acknowledgement that mental health is a problem and care is important • Support the idea of providing mental health care within the health center • Willingness to maintain a relationship with persons with mental illness • Belief that treating mental illness in the community would better integrate patients into regular life • Recommend that mental health screening should take place at each visit • Supported adopting a more tolerant attitude towards the mentally ill • In support of spending more tax money on the care and treatment of the mentally ill
		Beliefs that mental illness is more difficult to diagnose than other illnesses	
		Beliefs that traditional healers were more effective than modern medicine	
		Uncomfortable attending to mentally ill people	
		Beliefs that anyone who had mental health problems should be avoided	
		Beliefs that it is difficult to work with people with mental illness	
		Beliefs that people with mental illness should be kept behind locked doors and excluded from public offices	
		Patients respond to screening in a dishonest manner	
		Patients would not comply with the provider’s recommendations	
		Patients would not accept to receive the diagnosis or treatment at the primary care level	
		Legal liability for charting a wrong diagnosis	
		Unsatisfied with the level of knowledge in mental health	
		Do not regard managing mental illnesses as their primary role	
		Counseling left to the few specialists on ground which in their view tended to be unsuccessful	
		Negative attitudes towards mental health and mental disorders and limited appreciation of integration into primary health care	
	Motivation to change	Low interest in delivering mental health care	• Improved supply system of psychotropic medicines • Trust from clients • Ability to understand the patient in a more holistic way • Convenience of service provision • Willingness to screen for mental health problems
		Increased workload and limited time	
		Lack of mental health support both at community and district levels	
		Limited resources for service delivery	
		Clients attending many clinics leading to inconsistent management of health problems	
Health system constraints	Management and/or leadership	No in-service training in mental health care	• Team collaboration • Adequate record system • Connected primary care and mental health services • Improved training and recruitment of specialized and other allied health workers • Presence of communication between the services • Patient and provider education opportunities to increase patient awareness and screening
		No formal discussions about mental health disorders with higher level supervisors	
		Inadequate coordination between general health workers and mental health specialists	
		Inadequate support from the district medical team	
		Low prioritization of mental health care at the lower levels	
		Lack of knowledge about system structures and work processes	
		Inability of the health system to respond to the clients’ broader needs	
		Restriction on prescription of psychotropic medicines	
		Challenges managing outreach services	
		Lack of integrated health professionals’ timetables	
		Uncoordinated care planning	
		No clearly defined integrated clinic roles	
		Disjointed services within a decentralized system	
		Inadequate numbers of more diverse staff to serve the linguistic minority	
	Financial resources	Inequities in funding	• Separate mental health budget line within the Ministry of Health budget
		Lack of employee benefits	
		Lack of reimbursement for services	
		Uncertainty about continued funding for community programs/services	
		Mental health budget cuts	
		Insufficient insurance coverage to meet the treatment option	
		High cost of hiring nursing and support staff	

The barriers and facilitators mentioned under *providers of care* are divided into factors related to (i) attitudes regarding program acceptability, appropriateness, and credibility; (ii) knowledge and skills; and (iii) motivation to change. These factors are closely linked and are perquisite to behavior change. While those mentioned under *health systems constraints* were divided into factors related to management and/or leadership and financial resources.

#### Attitudes regarding program acceptability, appropriateness, and credibility

PCP attitudes regarding acceptability, appropriateness, and credibility towards integration of mental health services into PHC were the most highly cited domains in all the eligible studies except one [46]. The main barriers to integration of mental health services into PHC were (a) beliefs that mental illness was a strange behavior and more difficult to diagnose than other illnesses and that traditional healers were more effective than modern medicine practitioners, as such they (PHC) felt uncomfortable attending to mentally ill people [24, 43, 45, 49, 55, 56]; (b) beliefs that anyone who had mental illness should be avoided because it is difficult to work with such people, and so, they should be kept behind locked doors and excluded from the public [43, 47, 49, 50, 54, 55]; (c) that patients respond to screening in a dishonest manner, would not comply with the provider’s recommendations including accepting to receive the diagnosis or treatment at the primary care level, and that there was a likelihood of legal liability for charting a wrong diagnosis [41, 44, 53]; and (d) the PCPs were unsatisfied with the level of knowledge they had in mental health and did not regard managing mental illnesses as their primary role. They left counseling to the few specialists on ground, which in their view tended to be unsuccessful [24, 47, 55]. There were generally negative attitudes towards mental health and mental disorders and a limited appreciation of its integration into PHC [55]. The PCPs thought that integration of mental and physical health care was inappropriate and would potentially undermine the patient’s need for mental health care to be independently valued and explored [56].In addition, the PCPs thought that the public believes that specialized mental health services were not readily available in all health facilities [55], and they find it challenging to communicate to the mentally ill about the services offered [40].

On the other hand, the facilitative factors we identified related to PCP attitudes regarding acceptability, appropriateness, and credibility towards integration of mental health services into PHC were as follows: (a) some PCPs viewed mental illness like any other disease [49, 50, 55] that could be successfully treated [48], (b) there was support for providing mental health care within a health center [24, 47], (c) the use of motivational interviewing to help patients identify a problem and treatment options [45], (d) the recommendation that mental health screening should take place at each visit [53] with strict adherence to the standards of practice [46], (d) they supported adopting a more tolerant attitude towards the mentally ill in order to provide the best possible care [50], (e) the recognition that caring for patients with mental illness required specific skills and evidence-based treatments [45], (f) that treating mental illness in the community improved the integration of patients into regular life [48], (g) the increased access to and availability of mental health care [56], and (h) the support of spending more tax money on the care and treatment of the mentally ill [53].

In this review, all factors related to attitudes regarding acceptability, appropriateness, and credibility (SURE framework) were classified along the “motivation” domain in the COM-B theory of behavior change, because they speak to self-conscious intensions and beliefs, desires, impulses, inhibitions, drivers, and reflex responses [32].

When analyzed by setting (developed and developing countries), we found that most of the barriers related to attitudes regarding acceptability, appropriateness, and credibility towards integration of mental health services into PHC were from seven developing countries [24, 47, 49, 50, 53–55] and six developed countries [40, 41, 43–45, 56]. Facilitative factors were reported from seven developing countries [24, 47–50, 53, 55] and three developed countries [45, 46, 56]. Of the seven developing countries, six were from Sub-Saharan Africa (SSA) [24, 49, 50, 53–55] and one was from Asia [47]. These findings thus showed that these barriers and facilitators cut across the development divide. When analyzed according to PCP categories, nurses were the subject in 14 studies [19, 24, 40, 42, 44–46, 48–51, 53–56], doctors in 3 studies [26, 41, 47], and CHWs in 3 [40, 46, 50]. We found that the reported factors also cut across all the PCP categories.

#### Knowledge and skills

The barriers related to the PCP knowledge and skills in integration of mental health services into PHC were identified in 16 studies [24, 26, 40–42, 44, 45, 47–53, 55, 56] and they included (a) inability to diagnose and treat mental illnesses [24, 26, 47, 52, 53], with the associated excessive referrals [42]; (b) inability to identify either an antipsychotic or antidepressant medication [24]; (c) lack of knowledge regarding psychosocial interventions [52]; (d) inadequate training in the use of mental health screening tools and current evidence-based treatment [26, 40, 44, 45, 51, 53]; and (e) limited mental health awareness in the community [47]. In addition, there was lack of knowledge about health system structures and processes for management of mental health [52].

On the facilitative side, some studies highlighted perceived competence in mental health care [47], knowledge of mental disorder symptoms [44], and prior training in mental health [48].

In this review, all factors related to knowledge and skills (SURE framework) were classified along the “capability” domain in the COM-B theory of behavior change, because of the need for knowledge or skills and strength or stamina to engage in any mental processes [32].

In analyzing by setting, barriers related to PCP knowledge and skills with regard to integration of mental health services into PHC were mainly from developing countries [24, 47–53, 55], five of which were located in SSA [24, 49, 50, 53, 55]. On the other hand, the identified facilitators under this domain were in articles from two developing countries [47, 48] and from one developed country [44]. Basing on categories of PCPs, we found no difference in the reporting of the factors related to knowledge and skills.

#### Motivation to change

A total of 14 eligible studies [19, 24, 26, 40, 41, 43–45, 47, 52–56] highlighted the following motivation-related barriers to integration of mental health services into PHC: (a) low interest in delivering mental health care [24, 26], (b) increased workload and limited time [24, 26, 40, 41, 43, 44, 47, 52], (c) lack of mental health support both at community and district level [19, 26, 52–54], (d) limited resources for service delivery [40, 45], (e) ramifications of charting a diagnosis of mental illness or caring for patients with mental illness [26, 41], and (f) clients attending many clinics leading to inconsistent management of health problems [54].

On the facilitative side, factors that featured as motivation to PCPs included the following: (a) that general health workers were allowed to prescribe and administer psychotropic medicines [42], (b) an improved supply system of psychotropic medicines [55], (c) trust on the PCPs from clients [52], (d) the ability to understand the patient in a more holistic way [56], (e) convenience of service provision [40], (f) the willingness by PCPs to screen for mental health problems [44], and (g) community support and ownership [19].

In this review, all factors related to motivation to change (SURE framework) were classified along the “opportunity” domain in the COM-B theory of behavior change, because they involve time, resources, location, cues, physical affordance, interpersonal influences, and social and cultural norms that influence the way people think about things [32].

In analyzing by setting, barriers related to motivation to change of the PCPs with regard to integration of mental health services into PHC were from eight developed countries [19, 26, 40, 41, 43–45, 56] and six developing countries [24, 47, 52–55], four of which were in SSA [24, 53–55]. The facilitative factors were from four developed countries [19, 40, 44, 56] and two developing countries [52, 55]. In terms of categories, the barriers were reported across PCP categories while facilitators were reported in only articles that studied nurses.

#### Management and/or leadership

Sixteen studies [19, 24, 26, 40–46, 48, 51–55] presented barriers/facilitators related to management and/or leadership. The barriers we identified included (a) lack of in-service training in mental health care, coupled with no formal discussions about mental health disorders with higher level supervisors [24, 41], (b) inadequate coordination between general health workers and mental health specialists [41–43, 46, 52], (c) inadequate support from the district medical team [54], (d) low prioritization of mental health care at the lower levels [55], (e) lack of knowledge about system structures and work processes [52], (f) inability of the health system to respond to the clients’ broader needs [53], (g) restriction on prescription of psychotropic medicines [55], and (h) challenges managing outreach services [40]. In addition, there was lack of integrated health professionals’ timetables, uncoordinated care planning, no clearly defined integrated clinic roles, disjointed services within a decentralized system, [26, 43, 45, 46, 51, 53, 55], and inadequate numbers of more diverse staff to serve the linguistic minority [40].

On the other hand, the facilitative factors indicated by the PCPs included( (a) team collaboration with an adequate record system that is connected with primary care and mental health services [52], (b) improved training and recruitment of specialized and other allied health workers [55], (c) the use of stepped-care model (screening, therapeutic interventions, referrals to higher levels of care) [53], (d) the presence of communication between the various services [42], and (e) patient and provider education opportunities to increase patient awareness and screening [44].

In this review, the factors related to management and/or leadership (SURE framework) were classified in the “opportunity” domain [32]. The barriers related to management and/or leadership were from nine developed countries [19, 26, 40–46] and seven developing countries [24, 48, 51–55], while facilitative factors were from three developing countries [52, 53, 55] and two developed countries [42, 44].

#### Financial resources

Five studies [40, 43, 46, 51, 55] highlighted finance resource-related barriers that the PCPs face in regard to integrating mental health services into PHC. These included (a) inequities in funding [51], (b) lack of employee benefits [46], (c) lack of reimbursement for services [41], (d) uncertainty about continued funding for community programs/services due to cuts in the budgets for mental health services [40, 46], (e) insufficient insurance coverage to meet the treatment option [43], and (f) high cost of hiring nursing and support staff [40].

Only one study (in Uganda) reported financial resources as a facilitative factor where a separate budget line for mental health, within the Ministry of Health budget [55], was identified under this domain.

The factors related to financial resources (SURE framework) were classified in the “motivation” domain [32]. When analyzed by setting, the barriers identified were largely from three developed countries [40, 43, 46] and two developing countries [51, 55] while the facilitative factor was from a developing country [55].

## Discussion

**Fig. 2:**
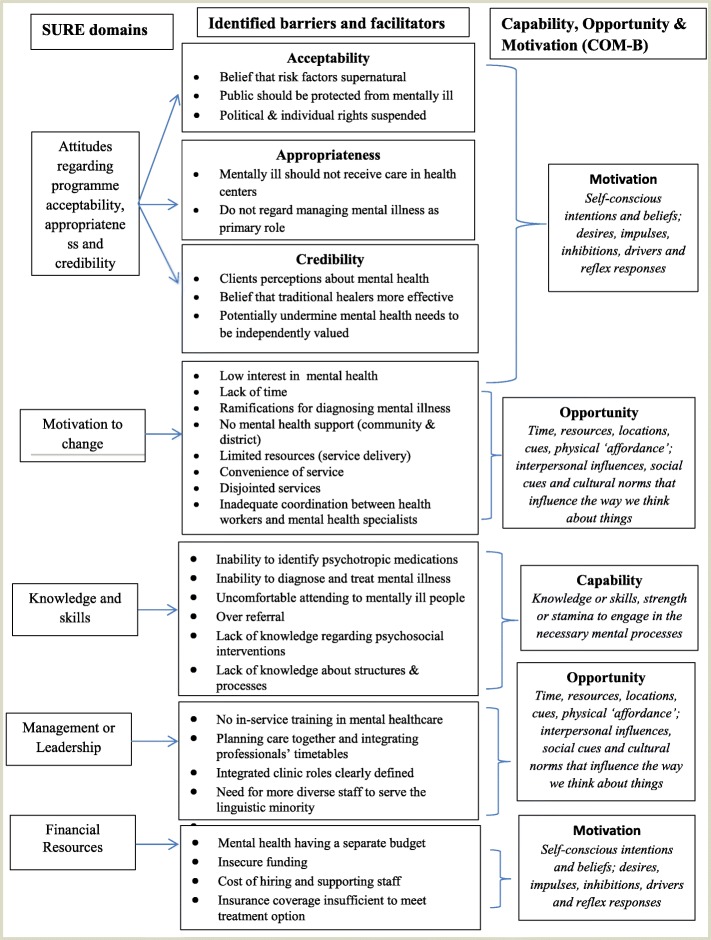
Linking SURE domains, identified barriers/facilitators, and COM-B domains

Methodologically, we used the SURE and COM-B as complementary frameworks as an innovation in the review. Whereas the SURE framework was validated for the identification of implementation factors, it does not provide practical steps to addressing them. The COM-B, on the other hand, provides a framework for understanding behavior of the PCPs towards the implementation of the option. Thus, we developed a model (Fig. 2) that linked the SURE domains, the identified barriers/facilitators, and COM-B framework of behavior change [33]. In reference to Fig. 2, classifying the identified barriers/facilitators along the COM-B domains provided more actionable options as opposed to when looked at solely along the SURE framework. The COM-B framework therefore provided better clarity in synthesizing findings from the review. We were not able to find a comparable study which used this methodology for identifying barriers and facilitators to integration of mental health services into PHC. The closest studies were a scoping review that examined the barriers and strategies to the implementation of guidelines but not COM-B related [58] and another that assessed the utility of a psychometric tool by general practitioners which utilized the COM-B framework [59].

From this review, it is evident that the policy of integration of mental health into PHC has been carried out in various countries, and our study confirms that there still exists poor or lack of integration of the option. We evaluated the existing literature through a rigorous process which involved reviewing qualitative, quantitative, and mixed methods studies using established tools to identify the existing factors, and we were able to identify important factors that need to be addressed.

In this study, we identified the key factors to integration of mental health services into PHC as PCP attitudes regarding program acceptability, appropriateness, and credibility; knowledge and skills; motivation to change; management and/leadership; and financial resources. While it was not our intention to carry out any comparative analysis between countries, we endeavored to show how the reviewed studies were distributed across the developmental divide. We have shown that the factors were cutting across the developmental divide (developed and developing countries) as well as across the PCP categories (nurses, doctors, and CHWs). These factors provide evidence that the mental health integration policy option is facing implementation challenges across the board thus requiring a consorted effort if they are to be addressed. The finding that while many studies reported financial resources as barriers, a study in an African developing country reported financial resources as a facilitative factor by setting aside a dedicated budget line for mental health within the Ministry of Health budget [55]. By this simple intervention, the country turned round a potential barrier into a facilitative factor, indicating that most barriers could be addressed using similar interventions.

The analysis from this review provides evidence to inform policy on the existing barriers and facilitators to the implementation of the mental health integration policy option. From the review, we found that despite the policy recommendations and implementation strategies provided, there were key barriers [24, 43, 45, 49, 55, 56] which if not considered will continue to compromise care for those with mental illness. On the other hand, there are also important key facilitators that could be optimized to advance integration of the mental health policy into practice.

### Implications

PCPs are the de facto gateway into general and specialized medical and mental health care. For mental health care, they are not only the first stop for the majority of patients with psychological symptoms, but for many, they may be the only stop [60, 61]. Understanding barriers and facilitators influencing the actions of PCPs using the COM-B system of behavior change provides a practical way of addressing barriers related to the integration of mental health care services into PHC which may improve the implementation of the policy option [62].

### Limitations

We did not include papers that were not published in English because of limited resources for language translation; it is therefore likely that some insights may have been missed out. Secondly, due to accessibility limitations, EMBASE was not searched hence potentially limiting our findings. In addition, some databases that we are not aware of may exist; hence, we may have missed out on relevant information. This manuscript is based on the data that was available to us.

## Conclusions

In this study, we identified the key factors to integration of mental health services into PHC as follows: PCP attitudes regarding program acceptability, appropriateness, and credibility; knowledge and skills; motivation to change; management and/leadership; and financial resources. These may have led to poor uptake of the mental health integration option. We therefore recommend studies to identify context-specific barriers the PCPs face with regards to integration of mental health services into PHC, from which relevant interventions can be developed.

## Additional files

Additional file 1: PRISMA checklist. (DOCX 29 kb)

Additional file 2: Search strategy. (DOCX 16 kb)

Additional file 3: SURE framework. (DOCX 19 kb)

Additional file 4: Study characteristics. (DOCX 70 kb)

Additional file 5: Risk of bias assessment. (DOCX 46 kb)

## Abbreviations

CASP: Critical Appraisal Skills Program
PHC: Primary health care
SURE: Supporting the Use of Research Evidence
WHO: World Health Organization
