# Supplementary material for: Barriers and facilitators to the integration of mental health services into primary health care: a systematic review
Source: Syst Rev. 2018 Nov 28;7:211. doi: 10.1186/s13643-018-0882-7 (PMC6264616; doi:10.1186/s13643-018-0882-7)
Supplement: Supplementary file 2 — Search strategy. (DOCX 16 kb) [file 13643_2018_882_MOESM2_ESM.docx]

**Additional file 2: Search Strategy**

**PubMed**

| Concept | Description of concept | Research Terms |
| --- | --- | --- |
| A | Mental Health | “Mental health”[Mesh] OR “Mental health[Text Word] OR Mental health[tiab] OR Mental health service*[Mesh] OR Mental health services*[tiab] OR Health services, mental[tiab] OR Health service, mental[Mesh] OR Service, mental health[Mesh] OR mental illness*[Mesh] OR mental disease*[Text Word] OR psychiatric condition*[Text Word] OR psychiatric diseases*[Text Word] OR psychiatric disorder*[Text Word] OR mental health service*[Text Word] OR mental health care[Text Word]))) |
| B | Integrate | Integrate OR integration[Text Word] |
| C | Primary Health Care | Primary Health care OR Primary Healthcare OR Community health care OR Community healthcare OR PHC OR Primary Care |

**PsycINFO**

| Concept | Description of concept | Research Terms |
| --- | --- | --- |
| A | Mental Health | Mental health OR Mental health OR Mental health OR Mental health services OR Mental health services OR mental illness OR mental disease OR mental diseases OR psychiatric conditions OR psychiatric condition OR psychiatric disease OR psychiatric diseases OR psychiatric disorder OR psychiatric disorders OR mental health services OR mental health care |
| B | Integrate | Integrat* |
| C | Primary Health Care | Primary Health care OR Primary Healthcare OR Community health care OR Community healthcare OR PHC OR Primary Care |

Search strategy « A » & « B » & « C »

**WHOLIS database**

| Concept | Description of concept | Research Terms |
| --- | --- | --- |
| Search A | Mental Health | (Mental health” OR Mental health service* OR mental illness* OR mental disease* OR psychiatric condition* OR psychiatric disease* OR psychiatric disorder* OR mental health care)  AND  Integrat*  AND  (Primary Health care OR Primary Healthcare OR Community health care OR Community healthcare OR PHC OR Primary Care) |

**OpenGrey database**

| Concept | Description of concept | Research Terms |
| --- | --- | --- |
| A | Mental Health | (“Mental health” OR “Mental health Mental health OR Mental health service* OR Mental health services*OR Health services, mental OR Health service, mental OR Service, mental health[Mesh] OR mental illness* OR mental disease* OR psychiatric condition* OR psychiatric disease* OR psychiatric disorder* OR mental health service* OR mental health care) AND (Integrate OR integration) AND (Primary Health care OR Primary Healthcare OR Community health care OR Community healthcare OR PHC OR Primary Care) |
