# Supplementary material for: Barriers and facilitators to the integration of mental health services into primary health care: a systematic review
Source: Syst Rev. 2018 Nov 28;7:211. doi: 10.1186/s13643-018-0882-7 (PMC6264616; doi:10.1186/s13643-018-0882-7)
Supplement: Supplementary file 4 — Study characteristics. (DOCX 70 kb) [file 13643_2018_882_MOESM4_ESM.docx]

**supportAdditional file 4: Characteristics of included studies**

CO= Clinical Officer, RN= Registered Nurse, MO= Medical Officer, HO=Health Officer, HNS= Health Nursing Supervisors, EHT= Environmental Health Technologist, FNP= Family Nurse Practitioners, VHA= Veteran’s Health Administration, PC=Primary Care, MH=Mental Health, CHO= community health officers, CEW= community extension workers, HA= health assistants, NP= Nurse practitioner, SHSM=Senior health service managers, SGM=Senior government managers, NL=Nursing leaders, MNGO=Manager, non-government organization, MHW=Mental health workers, GP=General practitioner, PS= Police superintendent, RCBO= Representative, community-based organization, PLWH=People Living with HIV, PWP=Psychological Well-being Practitioners

| **Lead Author, Year, Country and setting** | **Study Aim** | **Study design** | **Facility type** | **Participants/sample size** | **Data collection methods** | **Mental health type** | **Barriers and or enablers** |
| --- | --- | --- | --- | --- | --- | --- | --- |
| Cowan (2012) (1)  India (Developing country) | To examine the mental  health-related knowledge and attitudes of doctors providing  PHC in a rural area of Karnataka in Southern | Quantitative | Private and government PHC clinics | 59 Doctors | Survey questionnaire | Depression and Psychosis | **Barriers:** Limited training; belief that it is difficult to work with mental health patients; counseling left to specialist and tends to be unsuccessful; limited time with each patient; lack of diagnostic capacity; lack of awareness of mental health in the community; underestimate of the number of patients determined to have mental health problems;  **Enablers:** Self-perceived competence; agreement that mental health problems are common & need to be attended to; high level of interest in mental health |
| Ayalon (2015) (2)  Israel (Developed country) | To examine physicians’ perceived  barriers to the management of mental illness in  primary care settings in Israel | Qualitative | Public clinics | Physicians (family medicine, internal medicine,  general/no specialty) | Focus group discussions | Depression and Anxiety | Inadequate awareness, skills, knowledge, information or training; inadequate knowledge and training in diagnosis of mental illness in primary care; Limited or no interest in diagnosing depression and anxiety; some patients do not want their mental illness documented; ramifications of charting a diagnosis of mental illness; providers unsure of their role as providers of mental health; Limited time available; inadequate sources of informal (by family and friends) and formal support (by mental health providers) for patients and physicians alike; limited availability of primary care physicians; inadequate or absent support system impacted on physicians ability to treat the patient adequately; disjointed services; communication difficulties between physicians and mental health providers; stigma and need for confidentiality; scarcity of services; emotional ramifications of caring for patients with mental illness |
| Abera (2014) (3)  Ethiopia (Developing country) | To assess the perceived challenges and opportunities for integrating mental health care into a primary healthcare from the perspective of PHC workers in a regional zone in Ethiopia. | Quantitative cross-sectional survey with semi-structured interviews | Out-patient services with some in-patient provision for emergency deliveries | 116 nurses  29 HO  6 midwives | Questionnaire and a semi-structured interview guide | General mental health care | **Barriers:** Few with clinical attachment during pre-service training; No in-service training in mental health care; low interest in delivering mental healthcare; belief that traditional healers are more effective than modern medicine; belief that mentally ill persons should not receive care in the health centers; belief that treating persons with MNS would put others patients at a risk; inability to neither list a mental disorder nor its symptoms; belief that risk factors for mental illness are supernatural or spiritual factors; inability to identify either an antipsychotic or antidepressant medication; unavailability of medications to treat mental illness; shortage of examination space; lack of time to deliver mental healthcare; no formal discussions about mental health disorders with higher level supervisors  **Enablers:** acknowledgement that mental health is a problem and care is important; support the idea of providing mental health care within the health center; positive attitudes towards delivering mental health care; knowledge about the existence of effective medication; un-satisfaction with level of mental health knowledge; belief that in-service training is needed for health professionals to improve their knowledge |
| Winer (2013) (4)  Saint Vincent and the Grenadines (SVG) (Developing country) | To assess knowledge of and attitudes toward mental illness among primary care providers in SVG | Mixed methods | District hospital | 41 RN  2 MO  6 HNS  4 FNP | Questionnaire and structured interview guide | General mental health care | **Barriers:** Limited training and resources; knowledge base is not sufficient to comfortably provide treatment; inadequate transportation, medication, and manpower; limited communication with the Mental Health Centre, especially when patients are admitted or discharged; sporadic visits from the psychiatrist or the psychiatric nurse  **Enablers:** Prior training in mental health; perception that persons with mental illness can live in community; willingness to maintain a relationship with persons with mental illness; belief that people with mental illness deserve as much attention as people with physical illness; agreement that mental illness can be successfully treated; belief that treating mental illness in the community would better integrate patients into regular life; recommendation to create more robust local resources; recognition that caring for patients with mental illness requires specific skills and training; recommendation for periodic mental health clinics in the District Health Center; belief that weekly clinics could improve access to mental health care; providers hoped to increase family collaboration with the District Health Center team |
| Kapungwe (2011) (5)  Zambia (Developing country) | To explore health care providers’ attitudes towards people with mental illness within two  districts in Zambia | Quantitative | Government facility | 39 CO  28 RN  36 EN  4 EHT | Survey questionnaire | General mental health | **Barriers:** belief that mental illness is a strange behavior; political and individual rights of persons with mental illness should be suspended while on treatment; mentally ill patients should not be treated in the same health center as general patients; mentally ill people should not be allowed to work or to have children; individual rights of people who are mentally ill restricted; uncomfortable attending to mentally ill people  **Enablers:** Work experience; dealt with a mentally ill person since they started work; acknowledgement that mental illness is a serious problem; generally do not agree with handcuffing violent mental patients or detention in a solitary place |
| Fickle (2007) (6)  USA (Developed) | To provide a foundation for understanding current provider practices in relation to guidelines for collaboration | Qualitative | PC clinics- VHA | Physicians Psychologists Nurses | Semi-structured telephone interviews | Depression | **Barriers:** treatment or referral is based on comfort level with treating depression; no demonstration of integration of MH and PC services with shared responsibility for patients; no formal care coordination activities or involvement through case management; inadequate numbers of PC providers; over referral by PC providers; little evidence of PC collaboration in the treatment of depression; Physical distance between the two services  **Enablers:** mental health-primary care consultation exists at all sites; there is communication between the services |
| Mosaku (2017) (7)  Nigeria (Developing country) | to provide information on attitudes of PHC workers towards the mentally ill and determine factors that affect such attitudes, with a view to designing programs directed at achieving a positive change with the overall goal of improving the health of the public and  Nigeria as a Nation | Quantitative | primary health care centers | 67 CHO & CEW  3 doctors  24 nurses  6 HA | cross sectional survey | General mental health | **Barriers:** belief that the public should be protected; keep mentally ill behind locked doors; avoid anyone who has mental problems; that the mentally ill are a burden on society; that the mentally ill should be isolated; that anyone with a history of mental problems should be excluded from public office; that mental health facilities should be kept out of residential neighborhoods  **Enablers:** agreement that mental illness is an illness like any other; understanding that virtually anyone can become mentally ill and that the mentally ill should not be treated as outcasts; agreement that more tax money should be spent on the care and treatment of the mentally ill; that there is need to adopt a more tolerant attitude toward the mentally ill, that there is need to provide the best possible care for the mentally ill; mental health services should be provided through community based facilities |
| Martinez (2017) (8)  Mexico (Developing country) | Identifying barriers  from the perspective of clinic personnel to integrating  behavioral health services in community-based primary care  clinics in Mexico City | Qualitative | primary health care clinics | 5 physicians  2 nurses,  8 social workers,  4 psychologists  1 psychiatrist  1 dentist  4 administrative  staff | semi-structured interview | General mental health | **Barriers:** Service issues (Staff shortages, Resource shortages, Budgetary issues, Inequities in funding, Lack of employee benefits, Time management, Appointment restrictions, Service incongruence with needs of community, Poor health insurance coverage); Language or cultural issues (Stereotyping, Stigma, Behavioral health treatment beliefs); Care recipient characteristics (Perceptions regarding patient care, Low financial resources, Mental health); Knowledge and information issues (Medications, Lack of training and knowledge in service availability) |
| Barraclough (2016) (9)  Australia (Developed) | to describe a nurse practitioner-led primary healthcare rural mental health service and evidence of how the service was integrated with other services and the community | Qualitative | multi-function non-health nongovernment organization (NGO) | 1 NP  5 SHSM  3 SGM  2 NL  1 MNGO  6MHW  1 GP  1PS  1 RCBO | semi-structured interviews and a ‘stakeholder meeting’ | ‘Dual’ mental health and drug and alcohol (D&A) | **Barriers:** limited collaboration between health, community and welfare agencies; Service gaps for clients with ‘dual’, mental health and drug and alcohol (D&A); discord between the approaches of the mental health and D&A services for dual diagnosis clients; professional risks associated with the NP’s dislocation from the wider mental health team  **Enablers:** full-time mental health qualified NPs; respect for the NP; community support and ownership |
| Hill (2016) (10) USA  (Developed) | explore barriers and facilitators to mental health screening and treatment among women at a rural, primary care clinic in Appalachia | Qualitative | Rural Health Clinic | 4 Nurses | Semi structured interviews | depression, anxiety, substance abuse, and intimate partner  violence | **Barriers:** operational barriers (patients coming infrequently, limited time for patient visits, and need for better reimbursement); Mental Health Competence (Lack of conﬁdence, experience, or adequate training with mental health issues); Predicted Patient Reactions (beliefs that patients respond to screening in a dishonest manner, noncompliance with the provider’s recommendations); Patient Attitudes (frustration with patients who want a “quick ﬁx” from medications)  **Enablers:** Screening protocol in place; willingness to screen; knowledge of mental disorder symptoms; patient and provider education opportunities to increase patient awareness and screening |
| Athié (2016) (11) Brazil (Developing country) | To analyze the perceptions of health professionals and managers about the integration of primary care and mental health | Mixed methods cross-sectional study | Health districts | 42 health managers, GPs, psychologists and psychiatrists | Survey and interviews | General mental health | **Barriers:** Lack of access to services; Lack of knowledge about system structures and work processes; Constraints regarding institutional processes; Lack of human resources; excessive working hours; Lack of knowledge regarding psychosocial interventions; Integrating different professionals’ timetables; Low frequency of mental health support in the community  **Enablers:** more chances of starting treatment once at the facility; Trust from people that they take care of; Team Collaboration; adequate Record System; Connecting primary care and mental health services; Planning care together; Helping non-specialists managing mental health problems; Training to identify mental health problems; Narrowing the communication gap between different work processes; Favoring primary teams to diagnose and prescribe appropriate interventions; Improving access to cases of difficult adherence; Delivering mental health care in the community |
| Davis (2012) (12)  USA (Developed) | To describe the experiences of and barriers for pediatricians in Kentucky in providing behavioral/mental health (B-MH) services in primary care settings | Quantitative |  | 70 Pediatricians | Online Survey | General mental health | **Barriers:** inaccessibility of mental health professionals; lack of communication and collaboration; lack of knowledge; Concerns about legal liability; Concerns about patient safety; Lack of appropriate training; Lack of feedback from mental health specialists; Lack of information about mental health services and resources; Lack of reimbursement for services; Lack of time |
| Duffy (2017) (13)  Zimbabwe (Developing country) | Examine the acceptability and feasibility of an integrated mental health and HIV stepped-care approach in nurses (facility based), community health workers, and traditional medicine practitioners (community-based) in nine diverse urban and rural communities across  Zimbabwe | A three-phase Mixed methods design with a longitudinal  cohort | Facility and community based urban and rural communities | 325 nurses, community health workers, and traditional medicine practitioners in nine communities | - Systematic literature  review, and 17 semi structured  interviews  - Mental health and HIV integration at the site level with supportive supervision  - Mental health and HIV integration at the site and community level  with supportive  supervision | General mental health and HIV | **Barriers:** lack of psychosocial services; inability of the health system to respond to the clients’ broader needs; Transportation in cases where clients exhibited suicidal ideation; inability to link with family members and friends of clients for supportive follow-up care; hospitals disregarded referrals from traditional medicine practitioners; challenges using the screening tool; Provider discomfort in addressing the topic; concern that clients might be offended or fear persecution; the screening tool too lengthy to integrate into practice; using screening tools only on clients who appeared sad/worried or who mentioned recent alcohol use and/or smelled of alcohol  **Enablers:** Recommendation for mental health screens to take place at each visit; widespread feasibility and acceptability; stepped-care model of screening, therapeutic interventions, and referrals to higher levels of care; increased conﬁdence to provide mental health screening, counseling, and referrals for PLWH; screening tools easy to use |
| Henderson (2017) (14)  Australia (Developed) | Explore the capacity  of three rural communities in South Australia to deliver integrated mental health support for older people | Qualitative | Rural | health and social service providers | Semi- structured interviews | General mental health and old age | **Barriers:** gaps in service delivery related to accessing specialists; non separation in jurisdictional responsibility between the State Government, which manages mental health, and the Federal Government, which has primary responsibility for aged care; uncertainty about continued funding for ccograms and services; Fragmentation of service delivery between Federal and State Governments; State government retreating from primary care and social welfare activities; Reliance upon private service providers; public/private divide in creating joint plans; lack of sufﬁcient resources to take new community referrals; Budget constraints; Insecure funding; bureaucratization and formalization of relationships; centralization of administration of services; Strict adherence to the standards of practice |
| Henke (2008) (15)  USA | Examines primary care physicians' views on obstacles to providing depression care and CCM-based  interventions promoted to address those barriers | Qualitative | Rural/urban | Physicians | Interview guide | Depression | Belief that diagnosing depression is more difficult than diagnosing other illnesses; patient resistance to diagnosis and treatment; poor compliance due to stigma; mental health system is fragmented and difficult to access; reduced availability of specialists; inadequate coordination between physicians and mental health specialists; Insurance coverage insufficient to meet the treatment of patients with depression; Lack of mental health expertise; Competing demands |
| Jenkins (2013) (16)  Kenya | Explore health worker perspectives on the challenges posed to integration of mental health into primary care by generic health system weakness | Qualitative study from RCT participants | Clinical field setting | Nurses and clinical officers  -11 from the intervention group  -14 from the control group | Focus group discussions | General mental health | Mental illness stigmatized than HIV/AIDS or leprosy; difficulty reviewing each client adequately and discussing problems comprehensively because of high numbers; lack of support from the district medical team; difficulty obtaining feedback about the client from the specialist; poor compliance with prescribed medication, and difficulty giving sustained follow up to clients; Clients attend many health facilities, which militate’ against good consistent long term management of health problems; lack of appropriate medications in the health facilities; difficulties with transport hindered referral; Lack of mental health targets from the national to the community level; health providers threatened to manage violent patients |
| Kigozi (2009) (17)  Uganda  (Developing country) | Describe the opportunities for and challenges to the integration of mental  health into primary health care in Uganda | Qualitative |  | Nurses and teachers | Semi-structured interviews and focus group discussions | General mental health | **Barriers:** limited appreciation of integration; no clear demonstration in practice of mental health training skills; poor appreciation of role in caring for people with mental illness; do not regard managing people with mental illness as their primary role; understaffing; few experts to provide technical support and supervision; irregular support; negative attitude towards mental health and mental disorders; mental health care under-prioritized at the lower levels; supply of mental health drugs included on the essential drug list irregular; Restriction on prescription of psychotropic medicines by the PHC nurses; patients go to traditional healers or faith healers instead of the health facilities; public believes that specialized mental health services are not readily available in all health facilities  **Enablers:** inclusion of mental health in the National Minimum Health Care Package; mental health has a separate budget line within the Ministry of Health budget; good leadership in mental health; improved training and recruitment of specialized and other allied health workers; reviewed curricula for medical training to increase exposure to mental health issues; improved supply system of medicines enabling the provision of psychotropic medicines; general health workers allowed to prescribe and administer psychotropic medicines; considerable involvement of other players has facilitated the reduction of stigma and discrimination; existence of a decentralized health system; improved acceptance of mental health problems |
| Knowles (2015) (18)  UK (Developed country) | Trial of collaborative care which tested whether depression could be improved in people with long term conditions (LTCs) by integrating low-intensity psychological interventions within the context of routine primary care management of LTCs.  To explore perceptions  about the model from participants’ own viewpoint | Nested interview study within a trial | Primary care practices | -PWP  -PN  -GP | Semi-structured interviews | Depression and diabetes | **Barriers:** integration of mental and physical health care considered inappropriate and potentially undermined the patient’s need for their mental health condition to be independently valued and explored; integration of physical and mental health treatment resisted by patients (freedom encroached upon requiring them to talk about other factors, outside of their physical health)  **Enablers:** increased opportunities for care co-ordination and information sharing; enhanced confidence to manage mood problems in the context of complex physical symptoms; understanding the patient in a more holistic way; increased access to and availability of mental health care |
| Mesidor (2011) (19)  USA (Developed country) | To examine barriers and facilitators to accessing and providing comprehensive primary health care  for individuals with  serious mental illnesses | Qualitative study as part of a randomized trial | Behavioral health agency | Administrators, providers of  care, and single NP | Key informant interviews | Serious mental illnesses and serious chronic health problems | **Barriers:** high cost of hiring nursing and support staff; lack of insurance reimbursement for the services of the NP; mental health budget cuts; additional responsibilities to staff; limited staff competencies; the need for a more diverse staff to serve linguistic minority communities; challenges of communicating to providers and clients about the new services offered; space limitations; managing outreach services for those who are unable or have significant challenges getting to the agency; the impact of client non-compliance with treatment recommendations; failure to show for a scheduled appointment; lack of client engagement and motivation on how services are delivered  **Enablers:** access to a comprehensive insurance that pays for needed services; access to transportation services; a health care agency that does not turn individuals away for an inability to pay; Convenience of Services; Supportive Staff Assistance |
| Zubkoff (2016) (20)  USA | To explore perceptions about current substance use disorders (SUD) services and identify barriers and facilitators to providing evidence-based SUD treatments | Qualitative | Veteran Affairs facilities | Psychologists, registered nurses, NP, psychiatrists, and care managers | Semi-structured  Interview guide | Substance Use Disorders (SUD) | **Barriers:** unsure of the current evidence-based treatments for SUD; medication not viewed as a viable treatment option; limited stafﬁng; limited resources such as space; lack of knowledge to offer SUD treatment in the integrated clinic; discrepancies in familiarity with Drug Enforcement Agency requirements to prescribe certain medications in the integrated clinic setting; not feeling it is the focus of their work; feel inadequately trained to offer evidence-based SUD treatments  **Enablers:** openness to the idea of offering some type of SUD services; perceived need for additional training on the evidence-based treatments; Staff consistently valued evidence-based treatments and inquired about effective SUD treatments that were appropriate; open to brief interventions such as motivational interviewing to help patients identify a problem and to determine if the patient would like to pursue treatment options; perception that medication should be offered in conjunction with other services such as group therapy or care management; perceived to improve access to mental health services, staff felt a time delineated phone-based consultation might be a useful way to connect with peers trained in substance abuse treatment; clarity on the services to be provided; integrated clinic staff roles were clearly deﬁned; Staff were familiar with and clearly articulated regulatory requirements of the integrated clinic |

**REFERENCES**

1. Cowan J, Raja S, Naik A, Armstrong G. Knowledge and attitudes of doctors regarding the provision of mental health care in Doddaballapur Taluk, Bangalore Rural district, Karnataka. International journal of mental health systems. 2012;6(1):21.

2. Ayalon L, Karkabi K, Bleichman I, Fleischmann S, Goldfracht M. Barriers to the treatment of mental illness in primary care clinics in israel. Administration and Policy in Mental Health and Mental Health Services Research. 2015.

3. Abera M, Tesfaye M, Belachew T, Hanlon C. Perceived challenges and opportunities arising from integration of mental health into primary care: a cross-sectional survey of primary health care workers in south-west Ethiopia. BMC health services research. 2014;14:113.

4. Winer RA, Morris-Patterson A, Smart Y, Bijan I, Katz CL. Knowledge of and attitudes toward mental illness among primary care providers in Saint Vincent and the Grenadines. Psychiatric Quarterly. 2013;84(3):395-406.

5. Kapungwe A, Cooper S, Mayeya J, Mwanza J, Mwape L, Sikwese A, et al. Attitudes of primary health care providers towards people with mental illness: Evidence from two districts in Zambia. African journal of psychiatry. 2011;14(4):290-7.

6. Fickel JJ, Parker LE, Yano EM, Kirchner JE. Primary care--mental health collaboration: An example of assessing usual practice and potential barriers. Journal of interprofessional care. 2007;21(2):207-16.

7. Mosaku KS, Wallymahmed AH. Attitudes of primary care health workers towards mental health patients: A cross-sectional study in osun state, nigeria. Community mental health journal. 2016.

8. Martinez W, Galvan J, Saavedra N, Berenzon S. Barriers to Integrating Mental Health Services in Community-Based Primary Care Settings in Mexico City: A Qualitative Analysis. Psychiatric services (Washington, DC). 2017;68(5):497-502.

9. Barraclough F, Longman J, Barclay L. Integration in a nurse practitioner-led mental health service in rural Australia. The Australian journal of rural health. 2016;24(2):144-50.

10. Hill SK, Cantrell P, Edwards J, Dalton W. Factors influencing mental health screening and treatment among women in a rural south central Appalachian primary care clinic. The Journal of Rural Health. 2016;32(1):82-91.

11. Athie K, Menezes AL, da Silva AM, Campos M, Delgado PG, Fortes S, et al. Perceptions of health managers and professionals about mental health and primary care integration in Rio de Janeiro: a mixed methods study. BMC health services research. 2016;16(1):532.

12. Davis DW, Honaker SM, Jones VF, Williams PG, Stocker F, Martin E. Identification and management of behavioral/mental health problems in primary care pediatrics: perceived strengths, challenges, and new delivery models. Clinical pediatrics. 2012;51(10):978-82.

13. Duffy M, Sharer M, Cornman H, Pearson J, Pitorak H, Fullem A. Integrating Mental Health and HIV Services in Zimbabwean Communities: A Nurse and Community-led Approach to Reach the Most Vulnerable. The Journal of the Association of Nurses in AIDS Care : JANAC. 2017;28(2):186-98.

14. Henderson J, Dawson S, Fuller J, O'Kane D, Gerace A, Oster C, et al. Regional responses to the challenge of delivering integrated care to older people with mental health problems in rural Australia. Aging & mental health. 2017:1-7.

15. Henke RM, Chou AF, Chanin JC, Zides AB, Scholle SH. Physician attitude toward depression care interventions: implications for implementation of quality improvement initiatives. Implementation science : IS. 2008;3:40.

16. Jenkins R, Othieno C, Okeyo S, Aruwa J, Kingora J, Jenkins B. Health system challenges to integration of mental health delivery in primary care in Kenya--perspectives of primary care health workers. BMC health services research. 2013;13:368.

17. Kigozi FN, Ssebunnya J. Integration of mental health into primary health care in Uganda: opportunities and challenges. Mental health in family medicine. 2009;6(1):37-42.

18. Knowles SE, Chew-Graham C, Adeyemi I, Coupe N, Coventry PA. Managing depression in people with multimorbidity: a qualitative evaluation of an integrated collaborative care model. BMC family practice. 2015;16:32.

19. Mesidor M, Gidugu V, Rogers ES, Kash-Macdonald VM, Boardman JB. A qualitative study: barriers and facilitators to health care access for individuals with psychiatric disabilities. Psychiatric rehabilitation journal. 2011;34(4):285-94.

20. Zubkoff L, Shiner B, Watts BV. Staff perceptions of substance use disorder treatment in VA primary care–mental health integrated clinics. Journal of substance abuse treatment. 2016;70:44-9.
