# Supplementary material for: Barriers and facilitators to the integration of mental health services into primary health care: a systematic review
Source: Syst Rev. 2018 Nov 28;7:211. doi: 10.1186/s13643-018-0882-7 (PMC6264616; doi:10.1186/s13643-018-0882-7)
Supplement: Supplementary file 5 — Risk of bias assessment. (DOCX 46 kb) [file 13643_2018_882_MOESM5_ESM.docx]

**Additional File 5: Risk of Bias Assessment**

YES (Y), Can’t tell (X), NO (N) and Not Applicable (N/A)

| **Question** | **Abera (2014)** | **Athie (2016)** | **Ayalon (2015)** | **Barraclough (2016)** | **Cowan (2012)** | **Davis (2012)** | **Duffy (2017)** |
| --- | --- | --- | --- | --- | --- | --- | --- |
| Was there a clear statement of the aims of the research? | Y | Y | Y | Y | N/A | N/A | Y |
| Is a qualitative methodology appropriate? | Y | Y | Y | Y | N/A | N/A | Y |
| Was the research design appropriate to address the aims of the research? | Y | Y | Y | Y | N/A | N/A | Y |
| Was the recruitment strategy appropriate to the aims of the research? | Y | Y | Y | Y | N/A | N/A | Y |
| Were the data collected in a way that addressed the research issue? | Y | Y | Y | Y | N/A | N/A | Y |
| Has the relationship between researcher and participants been adequately considered? | Y | Y | Y | Y | N/A | N/A | Y |
| Have ethical issues been taken into consideration? | Y | Y | Y | Y | N/A | N/A | Y |
| Was the data analysis sufficiently rigorous? | N | Y | Y | Y | N/A | N/A | Y |
| Is there a clear statement of findings? | Y | Y | Y | Y | N/A | N/A | Y |
| 10.     Value of the research? (Contribution, new research areas, etc.) | Y | Y | Y | Y | N/A | N/A | Y |
| **CRITICAL APPRAISAL FOR QUANTITATIVE STUDIES** |  |  |  |  |  |  |  |
| **A.       SELECTION BIAS** |  |  |  |  |  |  |  |
| 1.        Are the individuals selected to participate in the study likely to be representative of the target population? | Y | Y | N/A | N/A | Y | Y | Y |
| 2.        Percentage of selected individuals agreed to participate indicated | Y | N | N/A | N/A | Y | N | N |
| **B.       STUDY DESIGN** |  |  |  |  |  |  |  |
| 1.        Indicate the study design | Y | Y | N/A | N/A | Y | Y | Y |
|        Was the study described as randomized? If NO, go to Component C | N | N | N/A | N/A | N | N | N |
|        If Yes, was the method of randomization described? |  |  | N/A | N/A |  |  |  |
|        If Yes, was the method appropriate? |  |  | N/A | N/A |  |  |  |
| **C.       CONFOUNDERS** |  |  |  |  |  |  |  |
| 1.        Were there important differences between groups prior to the intervention? | N/A | N | N/A | N/A | N | N | N |
| 2.        If yes, indicate the percentage of relevant confounders that were controlled (either in the design (e.g. stratification, matching) or analysis)? |  |  | N/A | N/A |  |  |  |
| **D.       BLINDING** |  |  |  |  |  |  |  |
| 1.        Was (were) the outcome assessor(s) aware of the intervention or exposure status of participants? | N/A | N/A | N/A | N/A | N | N | N |
| 2.        Were the study participants aware of the research question? | N/A | N/A | N/A | N/A | X | X | X |
| **E.        DATA COLLECTION METHODS** |  |  |  |  |  |  |  |
| 1.        Were data collection tools shown to be valid? | Y | Y | N/A | N/A | N | N | N |
| 2.        Were data collection tools shown to be reliable? | Y | Y | N/A | N/A | N | N | N |
| **F.        WITHDRAWALS AND DROP-OUTS** |  |  |  |  |  |  |  |
| 1.        Were withdrawals and drop-outs reported in terms of numbers and/or reasons per group? | N | N | N/A | N/A | N | N | N |
| 2.        Indicate the percentage of participants completing the study. (If the percentage differs by groups, record the lowest). | N | N | N/A | N/A | N | N | N |
| **G.       INTERVENTION INTEGRITY** |  |  |  |  |  |  |  |
| 1.        Percentage of participants who received the allocated intervention or exposure of interest | N | N | N/A | N/A | N/A | N/A | N |
| 2.        Was the consistency of the intervention measured? |  | N | N/A | N/A | N/A | N/A | N |
| 3.        Is it likely that subjects received an unintended intervention (contamination or co-intervention) that may influence the results? | N | N/A | N/A | N/A | N/A | N/A | N |
| **H.       ANALYSES** |  |  |  |  |  |  |  |
| 1.        Indicate the unit of allocation | N | N/A | N/A | N/A | N/A | N/A | N |
| 2.        Indicate the unit of analysis | N | Y | N/A | N/A | N/A | N/A | N |
| 3.        Are the statistical methods appropriate for the study design? | Y | N/A | N/A | N/A | Y | Y | X |
| 4.        Is the analysis performed by intervention allocation status (i.e. intention to treat) rather than the actual intervention received? | N | N/A | N/A | N/A | N/A | N/A | N |

| **Question** | **Fickel (2007)** | **Henderson (2017)** | **Henke (2008)** | **Hill (2016)** | **Jenkins (2013)** | **Kapungwe (2011)** | **Kigozi (2009)** |
| --- | --- | --- | --- | --- | --- | --- | --- |
| Was there a clear statement of the aims of the research? | Y | Y | Y | Y | Y | N/A | Y |
| Is a qualitative methodology appropriate? | Y | Y | Y | Y | Y | N/A | Y |
| Was the research design appropriate to address the aims of the research? | Y | Y | Y | Y | Y | N/A | Y |
| Was the recruitment strategy appropriate to the aims of the research? | Y | Y | Y | Y | Y | N/A | Y |
| Were the data collected in a way that addressed the research issue? | Y | Y | Y | Y | Y | N/A | Y |
| Has the relationship between researcher and participants been adequately considered? | Y | Y | Y | Y | Y | N/A | N |
| Have ethical issues been taken into consideration? | Y | Y | Y | Y | Y | N/A | Y |
| Was the data analysis sufficiently rigorous? | Y | Y | Y | Y | Y | N/A | Y |
| Is there a clear statement of findings? | Y | Y | Y | Y | Y | N/A | Y |
| 10.     Value of the research? (Contribution, new research areas, etc.) | Y | Y | Y | Y | Y | N/A | Y |
| **CRITICAL APPRAISAL FOR QUANTITATIVE STUDIES** |  |  |  |  |  |  |  |
| **A.       SELECTION BIAS** |  |  |  |  |  |  |  |
| 1.        Are the individuals selected to participate in the study likely to be representative of the target population? | N/A | N/A | N/A | N/A | Y | Y | N/A |
| 2.        Percentage of selected individuals agreed to participate indicated | N/A | N/A | N/A | N/A | N | N | N/A |
| **B.       STUDY DESIGN** |  |  |  |  |  |  |  |
| 1.        Indicate the study design | N/A | N/A | N/A | N/A | Y | Y | N/A |
|        Was the study described as randomized? If NO, go to Component C | N/A | N/A | N/A | N/A | Y | N | N/A |
|        If Yes, was the method of randomization described? | N/A | N/A | N/A | N/A | Y |  | N/A |
|        If Yes, was the method appropriate? | N/A | N/A | N/A | N/A | Y |  | N/A |
| **C.       CONFOUNDERS** |  |  |  |  |  |  |  |
| 1.        Were there important differences between groups prior to the intervention? | N/A | N/A | N/A | N/A | X | N | N/A |
| 2.        If yes, indicate the percentage of relevant confounders that were controlled (either in the design (e.g. stratification, matching) or analysis)? | N/A | N/A | N/A | N/A |  |  | N/A |
| **D.       BLINDING** |  |  |  |  |  |  |  |
| 1.        Was (were) the outcome assessor(s) aware of the intervention or exposure status of participants? | N/A | N/A | N/A | N/A | Y | N | N/A |
| 2.        Were the study participants aware of the research question? | N/A | N/A | N/A | N/A | N | N | N/A |
| **E.        DATA COLLECTION METHODS** |  |  |  |  |  |  |  |
| 1.        Were data collection tools shown to be valid? | N/A | N/A | N/A | N/A | N | Y | N/A |
| 2.        Were data collection tools shown to be reliable? | N/A | N/A | N/A | N/A | N | Y | N/A |
| **F.        WITHDRAWALS AND DROP-OUTS** |  |  |  |  |  |  |  |
| 1.        Were withdrawals and drop-outs reported in terms of numbers and/or reasons per group? | N/A | N/A | N/A | N/A | N | N | N/A |
| 2.        Indicate the percentage of participants completing the study. (If the percentage differs by groups, record the lowest). | N/A | N/A | N/A | N/A | N | N | N/A |
| **G.       INTERVENTION INTEGRITY** |  |  |  |  |  |  |  |
| 1.        Percentage of participants who received the allocated intervention or exposure of interest | N/A | N/A | N/A | N/A | N | N | N/A |
| 2.        Was the consistency of the intervention measured? | N/A | N/A | N/A | N/A | X | N | N/A |
| 3.        Is it likely that subjects received an unintended intervention (contamination or co-intervention) that may influence the results? | N/A | N/A | N/A | N/A | X | N | N/A |
| **H.       ANALYSES** |  |  |  |  |  |  |  |
| 1.        Indicate the unit of allocation | N/A | N/A | N/A | N/A | N | N | N/A |
| 2.        Indicate the unit of analysis |  |  |  |  | N | N |  |
| 3.        Are the statistical methods appropriate for the study design? | N/A | N/A | N/A | N/A | N/A | Y | N/A |
| 4.        Is the analysis performed by intervention allocation status (i.e. intention to treat) rather than the actual intervention received? | N/A | N/A | N/A | N/A | N | N | N/A |

| **Question** | **Knowles (2015)** | **Martinez (2017)** | **Mesidor (2011)** | **Mosaku (2016)** | **Winer (2013)** | **Zubkoff (2016)** |
| --- | --- | --- | --- | --- | --- | --- |
| Was there a clear statement of the aims of the research? | Y | Y | Y | N/A | Y | Y |
| Is a qualitative methodology appropriate? | Y | Y | Y | N/A | Y | Y |
| Was the research design appropriate to address the aims of the research? | Y | Y | Y | N/A | Y | Y |
| Was the recruitment strategy appropriate to the aims of the research? | Y | Y | Y | N/A | X | Y |
| Were the data collected in a way that addressed the research issue? | Y | Y | Y | N/A | Y | Y |
| Has the relationship between researcher and participants been adequately considered? | Y | Y | Y | N/A | X | Y |
| Have ethical issues been taken into consideration? | Y | Y | Y | N/A | Y | Y |
| Was the data analysis sufficiently rigorous? | Y | Y | Y | N/A | Y | Y |
| Is there a clear statement of findings? | Y | Y | Y | N/A | Y | Y |
| 10.     Value of the research? (Contribution, new research areas, etc.) | Y | Y | Y | N/A | Y | Y |
| **CRITICAL APPRAISAL FOR QUANTITATIVE STUDIES** |  |  |  |  |  |  |
| **A.       SELECTION BIAS** |  |  |  |  |  |  |
| 1.        Are the individuals selected to participate in the study likely to be representative of the target population? | N/A | N/A | Y | Y | Y | N/A |
| 2.        Percentage of selected individuals agreed to participate indicated | N/A | N/A | N | Y | N | N/A |
| **B.       STUDY DESIGN** |  |  |  |  |  |  |
| 1.        Indicate the study design | N/A | N/A | Y | Y | Y | N/A |
|        Was the study described as randomized? If NO, go to Component C | N/A | N/A | Y | N | N | N/A |
|        If Yes, was the method of randomization described? | N/A | N/A | Y |  |  | N/A |
|        If Yes, was the method appropriate? | N/A | N/A | Y |  |  | N/A |
| **C.       CONFOUNDERS** |  |  |  |  |  |  |
| 1.        Were there important differences between groups prior to the intervention? | N/A | N/A | X | X | N | N/A |
| 2.        If yes, indicate the percentage of relevant confounders that were controlled (either in the design (e.g. stratification, matching) or analysis)? | N/A | N/A |  |  |  | N/A |
| **D.       BLINDING** |  |  |  |  |  |  |
| 1.        Was (were) the outcome assessor(s) aware of the intervention or exposure status of participants? | N/A | N/A | Y | X | N | N/A |
| 2.        Were the study participants aware of the research question? | N/A | N/A | Y | X | N | N/A |
| **E.        DATA COLLECTION METHODS** |  |  |  |  |  |  |
| 1.        Were data collection tools shown to be valid? | N/A | N/A | X | Y | N | N/A |
| 2.        Were data collection tools shown to be reliable? | N/A | N/A | X | Y | N | N/A |
| **F.        WITHDRAWALS AND DROP-OUTS** |  |  |  |  |  |  |
| 1.        Were withdrawals and drop-outs reported in terms of numbers and/or reasons per group? | N/A | N/A | N | N | N | N/A |
| 2.        Indicate the percentage of participants completing the study. (If the percentage differs by groups, record the lowest). | N/A | N/A | N | N | N | N/A |
| **G.       INTERVENTION INTEGRITY** |  |  |  |  |  |  |
| 1.        Percentage of participants who received the allocated intervention or exposure of interest | N/A | N/A | N | N | N | N/A |
| 2.        Was the consistency of the intervention measured? | N/A | N/A | X | N | N | N/A |
| 3.        Is it likely that subjects received an unintended intervention (contamination or co-intervention) that may influence the results? | N/A | N/A | X | N | N | N/A |
| **H.       ANALYSES** |  |  |  |  |  |  |
| 1.        Indicate the unit of allocation | N/A | N/A | N | N | N | N/A |
| 2.        Indicate the unit of analysis |  |  | N | Y | N |  |
| 3.        Are the statistical methods appropriate for the study design? | N/A | N/A | N/A | Y | Y | N/A |
| 4.        Is the analysis performed by intervention allocation status (i.e. intention to treat) rather than the actual intervention received? | N/A | N/A | X | N | N | N/A |
